# Grain Boundary Plane Orientation Fundamental Zones and Structure-Property Relationships

**DOI:** 10.1038/srep15476

**Published:** 2015-10-26

**Authors:** Eric R. Homer, Srikanth Patala, Jonathan L. Priedeman

**Affiliations:** 1 Department of Mechanical Engineering, Brigham Young University, Provo, UT 84602, USA; 2 Department of Materials Science & Engineering, North Carolina State University, Raleigh, NC 27695, USA

## Abstract

Grain boundary plane orientation is a profoundly important determinant of character in polycrystalline materials that is not well understood. This work demonstrates how boundary plane orientation fundamental zones, which capture the natural crystallographic symmetries of a grain boundary, can be used to establish structure-property relationships. Using the fundamental zone representation, trends in computed energy, excess volume at the grain boundary, and temperature-dependent mobility naturally emerge and show a strong dependence on the boundary plane orientation. Analysis of common misorientation axes even suggests broader trends of grain boundary energy as a function of misorientation angle and plane orientation. Due to the strong structure-property relationships that naturally emerge from this work, boundary plane fundamental zones are expected to simplify analysis of both computational and experimental data. This standardized representation has the potential to significantly accelerate research in the topologically complex and vast five-dimensional phase space of grain boundaries.

Grain boundaries (GBs) in polycrystalline materials have a significant influence on numerous material properties^1^,^2^,^3^,^4^,^5^,^6^,^7^,^8^,^9^. Grain boundary engineering (GBE) has demonstrated that enhanced performance can be achieved when the population and connectivity of different GB types can be controlled^10^,^11^,^12^,^13^,^14^,^15^,^16^,^17^,^18^,^19^,^20^,^21^. The properties of GBs depend on the five crystallographic parameters, the misorientation (three) and the boundary-plane orientation (two), that classify the structure of GBs. However, most GB classification schemes ignore the role of the GB plane orientation and instead focus just on the three misorientation parameters, and sometimes reduce this to a single parameter, such as the disorientation angle. This focus on a reduced description is due in part to the size of the five-dimensional GB space^22^,^23^, and also due to the fact that remarkable improvements of properties have been realized for materials where the population of Σ3^n^ GBs can be controlled^12^,^17^. With the advent of advanced manufacturing techniques^24^ it will soon be possible to precisely control several microstructural features including texture, GB character and triple junction network distributions^25^. The simplified classification schemes that have served so well to date will need to be extended to capture the full five-dimensional GB character. This more complete description will enable microstructure-sensitive synthesis and GBE to be fully exploited in the design of next-generation engineering materials.

The importance of the complete GB character, in particular the plane orientation, has been emphasized in several recent experimental and computational investigations. For example, substantial variations in Chromium segregation have been observed for Σ3 GBs under irradiation, which is attributed to the GB plane orientation^26^. In Σ7 GBs of alumina, Matsunaga *et al*.^27^ have observed a strong dependence of the GB plane orientation on creep resistance. In Cu irradiated with He ions, the GB sink efficiency is shown to strongly depend on the overall GB character, which include both the misorientation and the plane orientation^28^. In Aluminum alloys, the precipitation of *β* phase at GBs has been proposed to depend strongly on the GB plane orientation^29^. King *et al*. have observed that the twin variants (Σ3^*n*^) don’t resist stress corrosion crack growth as well as expected, and the resistant GBs can only be identified by analyzing their complete character, including GB plane orientation^30^.

A significant obstacle in the discovery of reliable GB structure-property relationships in the five-dimensional phase space is the lack of a standardized representation of GB plane orientations. Even though recent experimental and computational advances facilitate the analysis of GBs in their full crystallographic detail, a consistent comparison of their properties and trends in distributions will be difficult without a standardized representation of GB plane orientation that naturally contains the crystallographic symmetry of the five-dimensional space.

The present work utilizes GB plane orientation fundamental zones (FZs) to demonstrate structure-property relationships that are profoundly influenced by the GB plane orientation. While the mathematical framework for GB plane orientation FZs has been published previously, the process for describing a given GB, or set of GBs, in the GB plane orientation FZ is included here to demonstrate the power of this technique to distinguish between GBs that might have similar crystallographic descriptions, but are very different boundaries. Using this standardized representation, structure-property relationships of energy, excess volume per unit GB area, and temperature-dependent mobility are examined, all of which show a strong dependence on GB plane orientation. Analysis of common disorientation axes even suggests broader trends of GB energy as a function of misorientation and GB plane orientation. The work concludes with a motivation for the adoption of a standardized representation of GB plane orientations.

## Results

### GB Plane Representation

To elucidate structure-property relationships as a function of the GB plane orientation, it is critical to first develop a consistent and unique description of the macroscopic parameters that describe the GB phase space. While several methods exist for the description of GB misorientation, we focus on the disorientation axis and angle, which describes misorientations in an irreducible space or FZ. This is particularly important because many GB misorientations have multiple axis-angle descriptions, depending on their symmetry, whereas in the FZ, only one description exists.

In contrast, no FZ descriptions for GB plane orientation have been adopted. GB plane orientation can be described by polar angles or other plane descriptors such as Miller indices, which are identical to the plane normal direction indices for cubic systems. A unique description of any given GB must take into account the fact that the GB plane has different indices when described from the two crystals on either side of the GB. The two descriptions are not independent, and hence describing a GB by both planes is redundant. To eliminate redundant information, only one plane need be specified, but this one plane should not suffer from the arbitrary selection of one of the two crystals as the reference frame. The GB plane FZ must also provide a common reference, since the symmetries in crystal orientations can make very different GBs appear to have identical descriptions, as will be demonstrated shortly.

**Figure 1:**
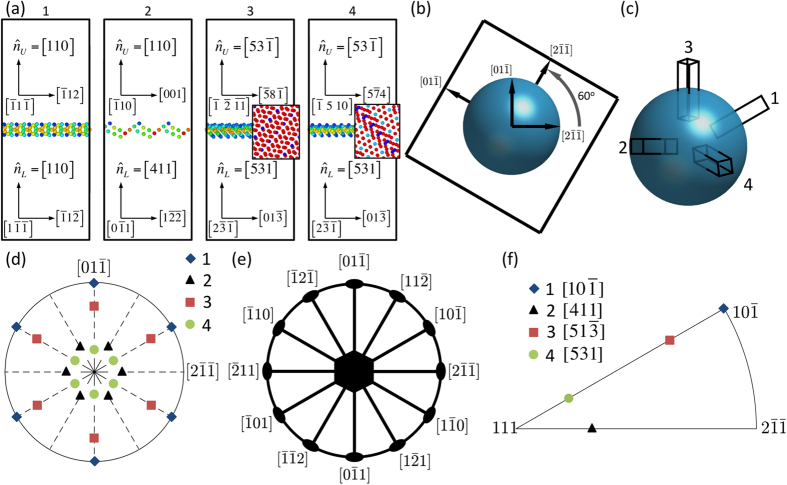
Determination of Boundary Planes in the FZ. Illustration of the process to determine a boundary plane orientation as defined in the FZ. (**a**) Comparison of four different incoherent Σ3 bicrystals with unique structure but common boundary plane definitions. Insets to bicrystals 3 and 4 show the structure in the GB plane to indicate their uniqueness. (**b**) Spherical Σ3 GB created by cutting out a sphere in a single crystal and then rotating the surrounding material about the [111] disorientation axis by 60°. (**c**) Orientation of bicrystals when brought into coincidence with the orientations of the crystals inside and outside the spherical GB. (**d**) Stereographic projection of symmetrically equivalent boundary planes that result from the symmetries of the CSL. (**e**) Projection of the D_6h_ boundary plane symmetries for the Σ3 misorientation, where bold lines are mirror planes, ellipses represent 2-fold symmetric rotations and the hexagon represents 6-fold symmetric rotations. (**f**) Stereographic projection of boundary plane normals in the FZ for the Σ3 misorientation.

As an example of the difficulty in uniquely and intuitively describing GB planes, consider four different Σ3 coincident site lattice (CSL) GBs, shown in Fig. 1a. The atomic structures of the GB are included, but only show atoms that are not surrounded by regular FCC atomic ordering. The GB normals for the bicrystals are given for both crystals, 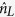 and 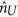, which are described in the lower (*L*) and upper (*U*) crystals, respectively. As can be seen, bicrystal 1 has the same indices for the normals in both crystals, 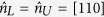. However, bicrystal 2 has different indices for normals as described from the two crystals, 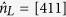 and 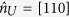. One might then wonder whether crystal *L* or *U* is the better or more appropriate descriptor of the GB. If 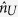 is used to parameterize the GB, the description 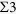 (110) will represent both the bicrystals 1 and 2. However, even if one consistently picks crystal *L* to serve as the reference crystal for GB plane normal, which would resolve the ambiguity between bicrystals 1 and 2, the consistent selection of one crystal is not sufficient. This is illustrated by comparing bicrystals 3 and 4. In both bicrystals, the GB plane indices are the same, 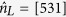 and 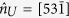. Yet, these are two unique bicrystals, as can be seen by the atomic structure in the GB plane shown in the insets to these two GBs in Fig. 1a. In fact, the exact rotations that result in bicrystals 3 and 4 are 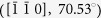 and ([210], 131.81°), respectively (both of which correspond to a Σ3 misorientation). While providing the complete axis-angle parameters of the misorientation in addition to the boundary-plane indices will resolve these ambiguities, such information is rarely provided and contains redundant information. Hence, it is desirable to attain a compact representation of the GB character, which does not suffer from above described ambiguities.

Patala and Schuh recently published a technique^31^ to take these seemingly confusing, contradictory and non-unique descriptions of GB plane orientations and provide a *unique*, relatively simple and intuitive description of a GB. Based on this work, there exists a FZ of GB plane orientations that takes into account all the symmetric descriptions of a given GB for a given disorientation and gives them a unique GB plane orientation descriptor.

While the work by Patala and Schuh details the mathematics for finding the GB plane orientation in the FZ^31^, the intuitive nature of the FZ and its application to a set of GBs is best understood by considering the following process. First, consider creating a Σ3 GB by taking a single crystal block of material, cutting out a sphere in the middle of that block and rotating the surrounding block about a given axis with respect to the spherical crystal in the middle. This is illustrated for the Σ3 GB in Fig. 1b, where the surrounding block is rotated by 60° about the [111] axis, the disorientation angle and axis for Σ3. This results in a single spherical GB, all of which belongs to a single misorientation, a Σ3 GB in this case. The GB also has plane orientations with normals that point in all directions. Since this spherical GB contains all possible plane orientations that could ever exist, including symmetric equivalents, this spherical GB becomes a convenient way by which to compare the bicrystals shown in Fig. 1a.

To take advantage of this intuitive description of a GB, the bicrystals must be brought into coincidence with the two crystals on either side of the spherical GB. To do this, we will define the interior spherical grain as reference crystal *L* and the surrounding grain as reference crystal *U*. Then, as before, each of the bicrystals will have the bottom crystal defined as crystal *L* and the top crystal as crystal *U*. All bicrystals will be rotated until they have their crystal *L* orientation brought into coincidence with the reference crystal *L* orientation. It is noted however, that at this point, crystal *U* in each of the bicrystals may or may not be in coincidence with the orientation of reference crystal *U*. One must then use the symmetry operators of the crystal to rotate the bicrystal as a whole until each bicrystal’s crystal *U* is in coincidence with the orientation of reference crystal *U*. These symmetry operators are to be applied in the reference frame of crystal *L*, such that it will leave bicrystal crystal *L* in coincidence with the reference crystal *L*. Following this process, the four Σ3 GBs shown in Fig. 1a, can now be seen as they emerge from specific positions of the spherical grain, as shown in Fig. 1c.

It is noted that the application of the symmetry operators, including switching the assignment of upper and lower grains in the crystal since the initial assignment is arbitrary, may provide more than one orientation of the bicrystal where both crystals are in coincidence with the reference crystals. Additional symmetrically-equivalent GB plane orientations may or may not appear depending on the symmetry of the bicrystal^31^,^32^. The additional symmetric descriptions of GB plane normal for the four bicrystals in Fig. 1a are plotted on a stereographic projection in Fig. 1d. One can see the symmetry in the stereographic projection, in Fig. 1d, that results from the symmetry of the bicrystal misorientation.

The projection of boundary plane symmetries for the Σ3 bicrystal is plotted in Fig. 1e. The FZ then, is a region of the spherical GB that contains all unique GBs, while all other regions of the sphere are symmetrical equivalents. The symmetries in the GB plane orientations for other misorientations and crystal systems are illustrated and described by Patala and Schuh^31^.

The GB plane normals for the four bicrystals shown in Fig. 1a, are plotted in the FZ in Fig. 1f. It can now be seen that the GB normal for bicrystals 1–4 in the FZ are 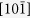, [411], 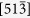, and [531] respectively. So even though bicrystals 1 and 2 both have some form of the [110] normal in crystal *L*, their unique descriptions are different. More importantly, bicrystals 3 and 4, which had the same direction indices for normals in crystals *L* and *U* for both bicrystals, now have unique normals that are different and accurately capture their unique GB plane orientation.

In order to interpret the FZs as given by Patala and Schuh^31^, it is helpful to point out an additional feature of this representation. By the inherent nature of the FZs, the boundaries to each of the symmetrically equivalent regions (24 in the case of Σ3) are defined by the point group symmetries of the dichromatic pattern (or the CSL), which in turn depends on its misorientation. This means that the FZ for each disorientation will be different. For example, the FZ for GB plane orientations of same disorientation axes but different disorientation angles will cover different regions of the sphere.

**Figure 2:**
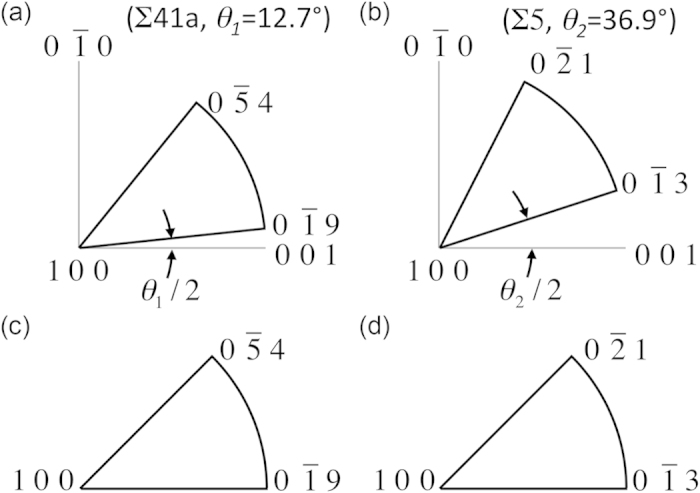
Representation of Boundary Plane FZs. Stereographic projections of FZs for the (**a**) Σ41a ([100] 12.68°) and (**b**) Σ5 ([100] 36.87°) CSL misorientations. To capture the inherent symmetry of the misorientations, the FZs are rotated from the [001] axis by half the disorientation angle, but cover a 45° swath of the sphere. (**c**,**d**) Standardized representation for the two FZs in (**a,b**) is accomplished by rotating the FZs down so that FZs of the same disorientation axis will appear identical.

To demonstrate the difference between two GBs with a similar disorientation axis but different disorientation angles we examine a Σ41a and a Σ5 GB, which are rotated by *θ*_1 _= 12.68° and *θ*_2 _= 36.87° about the [100] disorientation axis, respectively. The FZs for these two different disorientation angles are plotted in Fig. 2a,b, where it can be seen how they are rotated relative to the 〈100〉 directions. As detailed in the work of Patala and Schuh^31^, the two FZs are both rotated by half of their disorientation angle. The symmetries inherently captured by this region are illustrated by the fact that GBs with 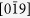 and 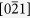 normals in the Σ41a and 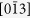 and 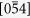 normals in the Σ5 are all symmetric tilt boundaries. It is important to note that these are symmetric tilt boundaries rotated about the disorientation axis [100], and not just any axis. These symmetric tilt GBs have been studied extensively and are created by rotating both crystals on either side of the GB by half the misorientation angle in opposite directions to get the full misorientation angle across the GB. It is also noted that the final normal bounding the FZ is [100] or the disorientation axis, and represents the pure twist boundary about the disorientation axis. The curved portion of the FZ corresponds to tilt boundaries about the disorientation axis. Even though the FZs are slightly rotated between GBs of same disorientation axis but different disorientation angle, these slight rotations capture the symmetries inherent to the underlying GBs.

To standardize presentation between FZs, they are always rotated down, as in Fig. 2c,d, such that the FZs appear similar for same disorientation axes. The formulas for selecting these axes that capture the natural symmetry of the FZ for a given disorientation axis and angle are provided by Patala and Schuh^31^.

It is noted that these descriptions focus on the GB plane orientation as described from only one crystal. This is emphasized as a feature of the standardized representation because it is simple and the description from the second crystal is redundant. However, supplemental Fig. S1 details the process for identifying the FZ as described in both crystals.

### GB Plane Orientation Property Trends

While it has always been known that GB plane orientation plays an important role in GB properties, the ability to plot these properties in the FZ provides new insights into trends of GB energy, mobility and excess volume per unit GB area.

**Figure 3:**
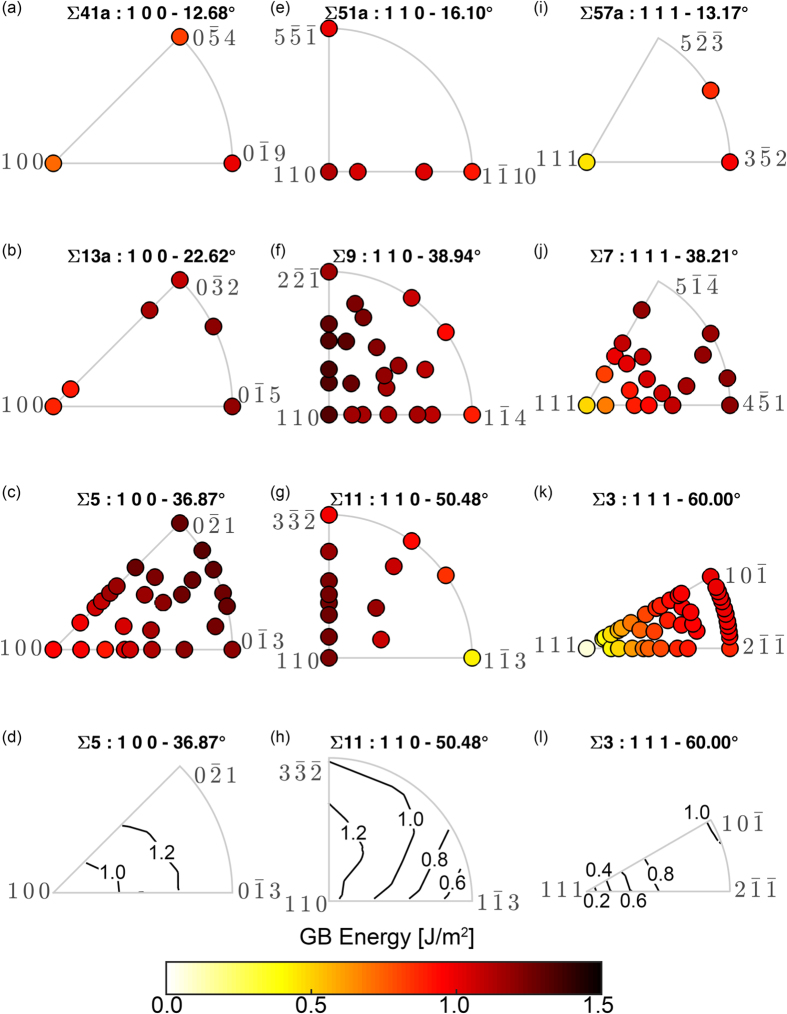
GB Energy structure-property relationships. Energy for GBs of 9 different CSL types plotted in their FZs and organized by common disorientation axes. The three columns show disorientations along the [100], [110], and [111] axes, in (**a**–**c**), (**e**–**g**), and (**i**–**k**), respectively. Contour plots of (**d**) Σ5, (**h**) Σ11, and (**l**) Σ3 show smooth variation of GB energy in the FZ.

GB energy, in particular, demonstrates the remarkable role of GB plane orientations. Figure 3 shows the GB plane FZs for 9 different CSLs. The GB energy data for each of the plots comes from the set of 388 Nickel GBs created by Olmsted *et al*.^33^ These FZs are organized by common disorientation axes, with the three columns showing similar axes of [100], [110], and [111], respectively. In each column, the CSLs are ordered by increasing disorientation angle from top to bottom. Due to the common disorientation axes, the FZs of each column are similar. Though it is noted that the Σ3 FZ is half the size of the other [111] disorientation axis CSLs due to its additional symmetry.

Since lower disorientation angles typically have higher Σ values, and higher Σ values tend to have larger GB areas, the size constraints of the GBs originally constructed by Olmsted typically limit the number of GB plane orientations for these higher Σ CSLs^33^. As such, we first direct our attention to the low Σ GBs, where numerous GB plane orientations provide a better understanding of how GB energy varies over the entire FZ. Though it may not be surprising, it is certainly notable that the GB energy varies smoothly in the Σ3, Σ5, Σ7, Σ9, and Σ11 CSLs.

In examining the Σ3 ([111], 60°), Σ5 ([100], 36.87°), and Σ7 ([111] 38.21°) FZs further, it is noted that the GB energy is lowest at the plane with indices equal to the disorientation axis and shows a near monotonic increase for normals moving away from the disorientation axis. This is illustrated in a contour plot of GB energy for Σ3 and Σ5 in Fig. 3l,d, respectively. In contrast, the Σ9 ([110], 38.94°) and Σ11 FZs ([110], 50.48°) have the highest GB energy at plane indices of 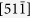 and 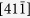, respectively, although these GB energies are very close in value to the energy of the plane with indices equal to the 110 disorientation axis (less than 25 mJ/m^2^ difference in both cases). The decrease in energy away from these highest energy indices is not entirely monotonic but close to a monotonic decrease. This decrease in energy is illustrated in a contour plot of GB energy for Σ11 in Fig. 3h.

These trends of the highest and lowest values of GB energy in the FZ appear to be similar for misorientations of similar disorientation axis. For example, if one examines the Σ13a ([100], 22.62°) FZ, there are considerably fewer normals than the Σ5 FZ, although the ones that are present support the monotonic increase in energy away from the plane normal equal to the disorientation axis. For the Σ41a ([100], 12.68°) FZ, there are only 3 GB plane orientations at the vertices of the FZ. While there is limited data, for Σ13a and Σ41a, to indicate how GB energy evolves as the plane orientation is varied, the plane normal equal to the disorientation axis has the lowest energy and the two plane normals at the vertices of the curved FZ boundary are higher in energy than the disorientation axis plane normal.

**Figure 4:**
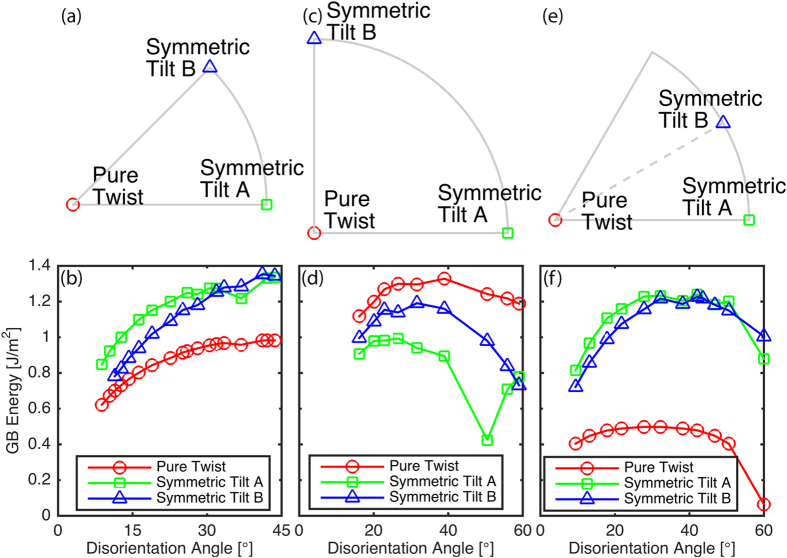
Structure-Property Relationships Across Common Disorientation Axes. Identification of pure twist and (A and B) symmetric tilt GBs in the (**a**) [100], (**c**) [110], and (**e**) [111] disorientation axis FZs, respectively. Trends of the pure twist and (A and B) symmetric tilt GBs across a range of disorientation angles in the common disorientation axes of (**b**) [100], (**d**) [110], and (**f**) [111], respectively.

There are additional [100] disorientation axis CSLs that have been simulated in the set of 388 GBs examined by Olmsted *et al*.^33^ Unfortunately, most of these additional CSL GBs only have 3 unique GB plane orientations, like the Σ41a. And, much like the Σ41a, the planes that do exist generally have plane normals at the vertices of the FZ. As described above, these vertices are special because the boundary plane with normal equal to the disorientation axis is a pure twist about that disorientation axis and the boundary planes with normals at the ends of the curved boundary of the FZ are symmetric tilt GBs. This is illustrated in Fig. 4a, and the two symmetric tilt GBs are distinguished as A and B for identification purposes. To compare the available data across all the [100] disorientation axis CSLs, we examine similar GBs across multiple disorientation angles. In particular, we select the pure twist and symmetric tilt (A and B) GB plane orientations across all the simulated [100] disorientation axis CSLs and plot their GB energy as a function of the disorientation angle in Fig. 4b. It can be seen that the pure twist GB is consistently the lowest energy, the symmetric tilt A has the highest energy for low disorientation angles and then both symmetric tilts are similar in energy for high disorientation angles. The different energy curves are smooth, with the exception of a small cusp in energy at the Σ5 disorientation angle of 36.87°. According to the data, the energy also shows a near monotonic increase from pure twist to the symmetric tilts, which suggests that energy for [100] disorientation CSL GBs is likely to be bounded by the pure twist and the higher of the two symmetric tilt GBs.

The [111] disorientation axis CSLs behave in a similar fashion to the [100] disorientation axis CSLs. As noted earlier, the Σ3 and Σ7 FZ energies show a monotonic increase away from the boundary plane with normal equal to the disorientation axis. For the Σ57a CSL, there are only boundary planes with normals at the disorientation axis, and at the lower vertex and the midpoint of the curved boundary. While there are only three data points available for the Σ57a ([111], 13.17°), the energies appear to follow the same trend as the Σ3 and Σ7 FZs. Again, there are additional [111] disorientation axis CSLs, but most only have boundary plane normals at the same points as the Σ57a. As indicated in Fig. 4e, these points correspond to the pure twist about the disorientation axis, a symmetric tilt boundary at the bottom vertex of the curved FZ boundary denoted as A, and another quasi-symmetric tilt boundary at the midpoint of the curved FZ boundary denoted as B. Since the B GBs include an additional stacking inversion across the GB, they are referred to as quasi-symmetric tilt GBs^34^,^35^. Figure 4f plots the energies for the pure twist and symmetric tilt GBs as a function of disorientation angle. Once again, the pure twist is the lowest energy GB, although in this case this energy is lower by a much larger margin than the [100] disorientation axis CSLs. For low disorientation angles, the symmetric tilt A GBs are the higher energy boundaries, but then at higher disorientation angles, both symmetric tilt A and B GBs have similar energies, and then at the highest disorientation angle, the quasi-symmetric tilt B GB is higher in energy. The curves are again smooth and the Σ3 appears to be a cusp in the curves.

Finally, the [110] disorientation axis GBs contrast the [100] and [111] CSL GBs in that the highest energy is usually near the [110] disorientation axis normal. As noted previously, the highest energy actually occurred at plane normals of 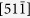 and 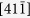 for the Σ9 and Σ11 CSLs, respectively. Unfortunately, none of the additional simulated [110] disorientation axis CSLs have normals that would follow the 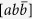 trend to confirm that this consistently leads to the highest GB energy. However, since this energy was consistently close to the energy of the [110] disorientation axis normal, and decreased for boundary plane normals moving away, we can still plot the general trend as was done for the [100] and [111] disorientation axis CSLs. As indicated in Fig. 4c, the vertices of the FZ correspond to a pure twist about the disorientation axis normal and symmetric tilt boundaries at the curved FZ boundary, denoted as A and B. Figure 4d plots the energies for the pure twist and symmetric tilt GBs as a function of disorientation angle. The pure twist is consistently higher than the symmetric tilts, although this difference increases at higher disorientation angles. The symmetric tilt A GBs have the lowest energy for all but the highest disorientation angle, with a significant cusp for the Σ11 at 50.48°.

**Figure 5:**
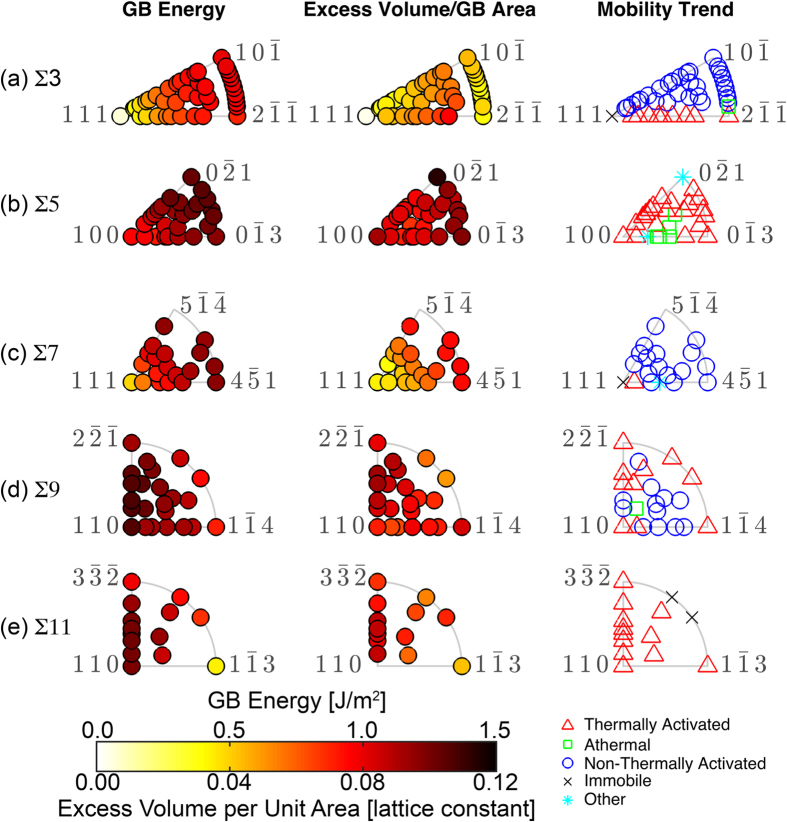
Various Structure-Property Relationships for Low-Σ CSLs. GB energy, excess volume per unit GB area, and temperature-dependent mobility trend plotted in the FZ for the (**a**) Σ3, (**b**) Σ5, (**c**) Σ7, (**d**) Σ9, and (**e**) Σ11 CSLs, respectively.

In contrast to GB energy, simple structure-property relationships over the GB plane FZs for other GB properties are not immediately obvious. In this examination, we focus just on the lowest CSLs, Σ3, Σ5, Σ7, Σ9, and Σ11, since there are sufficient GBs to discern possible trends. Figure 5 plots GB energy, excess volume per unit of GB area, and temperature dependent mobility trend in the FZ for each CSL. The excess volume per unit area for each CSL generally correlates well with the GB energy trends. This correlation, between properties has been noted in previous work, though there was no indication in the previous work that GB plane orientation played a role^33^. However, even though this correlation exists, there are notable exceptions. For example, the Σ3 GBs show an initial increase in excess volume as the normals deviate from the pure twist, but the excess volume per unit GB area drops back down at the curved FZ boundary.

In contrast to the excess free volume, the mobility trends for the CSLs in Fig. 5 do not appear to correlate with any other property. However, the mobility trends do appear to be highly dependent upon the GB plane orientation. In the initial publication of the mobility trends, there did not appear to be any crystallographic reason for why one boundary would exhibit a thermally activated mechanism as opposed to a non-thermally activated mechanism. This was particularly true of the Σ3 GBs, of which a high percentage exhibited the thermally damped motion mechanism, a non-thermally activated mechanism where mobility is inversely proportional to temperature, *M *= 1/*T *+ constant. When the mobility trends for the Σ3 were finally plotted in the GB plane orientation FZ, the authors could not have been more surprised by the result. All of the thermally activated GBs have normals that fall between the [111] disorientation axis, which is the immobile coherent twin, and the 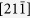 axis. All other boundary planes exhibit some form of non-thermally activated mobility trend, either thermally damped or athermal mobility. While the mobility trends for the Σ3 GBs have clear dependence upon the GB plane orientation, the exact reason for this is not presently known and is the subject of ongoing research.

The Σ5 mobility trends are mostly thermally activated, although there are a number of GBs that are athermal, whose normals are located in the lower center of the FZ. Finally, there are two other GBs that behaved in a fashion that could not be classified in the general trends of thermally activated or non-thermally activated, and as such are noted as “other”. The majority of the Σ7 GBs are non-thermally activated, with the exceptions having plane orientations close to the [111] axis. The Σ9 GBs exhibit the full range of mobility trends and there is not any clear boundary plane orientation dependence. Finally, the Σ11 GBs are either thermally activated or immobile, exhibiting no non-thermally activated temperature-dependent mobility trends.

These mobility trends do not appear to correlate in a one to one fashion with either GB energy or excess volume per unit GB area. Nevertheless, there is a clear dependence of these trends on GB plane orientation, which warrants further study.

### Analysis of GB Property Trends

Analysis of GB energy across the [100], [110], and [111] disorientation axes indicate promising structure-property relationships. These are divided mainly into two different trends. The first is that, for each disorientation axis, the GB energy for different boundary plane orientations appear to be increasing or decreasing nearly monotonically across the FZ. The variation in energy is generally smooth and the twist and symmetric tilt GBs about the disorientation axis are typically the maximum or minimum values of energy in any one FZ. Second, similar disorientation axes have similar GBs that exhibit the maximum and minimum values across a range of disorientation angles. Together, these two trends suggest that with limited knowledge about GBs with [100], [110], and [111] disorientation axes, one could potentially estimate energy for just about any GB with the disorientation axis along [100], [110], or [111], no matter the disorientation angle or boundary plane orientation.

The strong structure-property relationships in the [100], [110], and [111] disorientation axis GBs are likely the reason that the GB energy function for FCC metals developed by Bulatov *et al*. works so well^36^. Their basis functions interpolate GB energy values from the [100], [110], and [111] misorientation axes and use the same set of 388 GBs. Based on the evidence provided here, the energies estimated by their GB energy function for just about any [100], [110], and [111] disorientation axis GB is likely to be highly accurate. The present work utilizes the natural symmetries to reduce the representation to a FZ, though it is noted that Bulatov *et al*. also observe continuous cusps in the energy that are likely indicative of broader structure-property relationships that will become evident with additional data. Finally, Bulatov *et al*. note that their GB energy function has only two material specific parameters. Thus, the trends found here are also likely valid across a range of FCC metals, and will scale with just a few material specific parameters.

The GBs that belong to the [100], [110], and [111] disorientation axes account for nearly 60% of the 388 GBs. Unfortunately, these three axes represent a very small fraction of the disorientation fundamental zone, meaning that the five-parameter GB space remains largely unexplored. The remaining 40% of the 388 GBs are spread over various disorientation axes whose GB plane FZs are not as small as the higher symmetry [100], [110], and [111] disorientation axis FZs. As such, it is difficult to extract any additional trends on GB energy from these remaining GBs.

In the low Σ CSLs, the property of excess volume per unit GB area shows a clear dependence on GB plane orientation but the exact reason for the variation is not immediately obvious. This is an interesting observation because both the GB energy and the excess volume per unit area are dependent upon the structure of the atoms at the boundary. However, it appears that the GB energy manages to vary smoothly while the excess volume per unit area is more sensitive to the structure at the GB. For example, in the tilt GBs on the curved portion of the Σ3 FZ, it is not surprising that the excess volume per unit GB area is low for the highly ordered tilt GB structures. At the same time, it is notable that these highly ordered structures do not correspond to lower values in GB energy. There is significant literature on correlations between GB structure, excess volume, and energy, with mixed agreement between theory and findings^34^,^37^.

In examining the mobility of the low Σ CSLs, it is noted that it does not correlate with the GB energy in the GB plane FZ; Olmsted *et al*. also failed to observe this correlation in their examination of the 388 GBs^38^. Nevertheless, these low Σ CSLs show strong dependence on GB plane orientation for the temperature-dependent mobility. Similar to the likely important role of structure in excess volume per unit area, GB mobility is known to be influenced by the atomic structure^39^; shear coupling demonstrates this structural dependence particularly well^35^,^40^,^41^. Unfortunately, examination of the 388 GBs in their respective FZs yields no observed trends on GB plane orientation. However, previous examination of these 388 GBs demonstrated shear coupling in many symmetric tilt GBs^35^, which populate the corners of the [100], [110], and [111] FZs. As a result, there may be some broader shear coupling trends that may emerge as more data is simulated, though they are not obvious in the present work.

## Discussion

GB plane orientation is an important determinant in character that is frequently ignored due in part to the complexity of the five-dimensional GB space as well as the lack of a standardized method for providing unique descriptors or representations of GB plane orientation. To continue advancing GB science, the authors believe a standardized representation of GB plane orientations needs to be adopted. The power and utility of misorientation FZs (the disorientations) have already been established and are common-place in the analysis of GB distributions and properties. The recent publication by Patala and Schuh provides the necessary tools to solve the standardized representation problem^31^ and the present work demonstrates the power of standardized descriptors to compare multiple GBs and thereby extract structure-property relationships for GBs.

Traditional methods of representing the full GB character typically invoke the tilt or twist character of the GB. While this is a useful criterion, the *same physical GB* may be expressed by multiple descriptions depending on the parameters used to describe the GB. For example, the 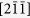 GB in Fig. 3k can be described as a 〈111〉 symmetric tilt (60° rotation, with each crystal rotated by half that angle) or as a 〈110〉 symmetric tilt (70.5° rotation, with each crystal rotated by half that angle). As a result, this GB often shows up in both [110] and [111] symmetric tilt GB plots^35^. Similarly, a Σ3 GB with plane orientation 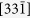 is an asymmetric tilt GB if Σ3 is expressed along the 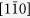 axis and a general (with no-tilt character) GB if the same misorientation is expressed along the [111] axis. However, the fact that different symmetrically equivalent parameters are used should not necessarily change any physical property or the structure of the GB. Other GB description techniques suffer from the same problems. For example, describing a GB by listing the orientation matrices for both crystals can suffer from the problems illustrated in Fig. 1, where the lack of a common reference frame complicates comparisons of GBs that might otherwise appear identical.

With the advent of high-throughput simulation and experimental techniques for the characterization of the complete five-dimensional crystallographic space of GBs, a standardized representation for the GB character provides the greatest opportunity to uniquely identify GBs and their contribution to measured properties. Electron backscatter diffraction (EBSD) methods are an excellent example of this as a lot of data is extracted in each scan. With proper representation, one could move beyond the misorientation-only description of the GBs, as is currently done. Standardized representation may overcome the fact that EBSD methods only extract four of the five GB parameters, leaving the boundary plane orientation distribution unresolved. Even if this last degree of freedom remains unknown, the possible property values the GB could exhibit could be fairly easily estimated from boundary plane FZ plots; the possible values would be a linear projection across the boundary plane FZ.

**Figure 6:**
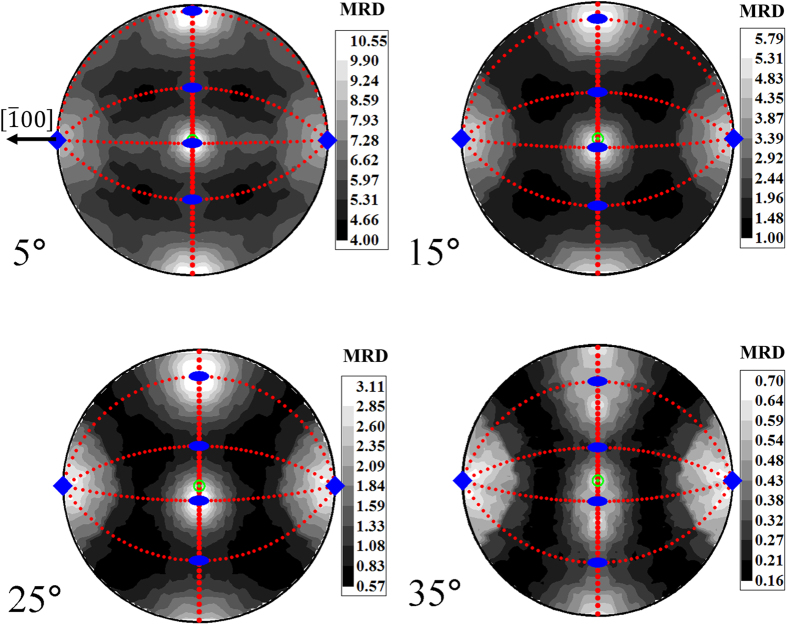
Overlaid Symmetries in Experimental Interface Distributions. An interface distribution from Rohrer *et al*.^42^ overlaid with the bicrystal symmetries of the corresponding misorientations ([001], (5^o^, 15^o^, 25^o^, 35^o^)). Note that the mirror planes (dashed red lines) capture the symmetries present in the distribution. Also note the natural rotation of the symmetries due to the increased misorientation angle. Finally, note that only a single region or FZ in any one graph is needed to describe the entire distribution, all other information is redundant. Original image from Rohrer *et al*^42^ © Carl Hanser Verlag, Muenchen is used with permission.

A standardized format for the representation of GB plane orientations is also essential for investigating the statistical distributions of GB populations in the full five-dimensional crystallographic phase space. For example, Rohrer *et al*.^42^ and Patala and Schuh^43^ plot experimental and model distributions of GB-plane orientations along a fixed disorientation axis. The evolution of the intensities gradually changes with the disorientation angles and is seemingly complex. However, if the FZs are super-imposed on the intensity plots (shown in Fig. 6), the trends and their corresponding symmetries are obvious. In the work of Rohrer *et al*., the intensity of the low-index [001] and the surrounding planes is high^42^. The other intensities observed on the sphere are symmetrically equivalent to these planes. As the disorientation angle is changed, the FZ gradually rotates as shown, for example, in Fig. 2. The edges of the FZ represent mirror-planes and, therefore, the intensities exhibit a mirror symmetry across the edges of the FZ. As the position of the FZ varies gradually, the mirror-image of the high intensity [001] planes also gradually shifts. Therefore, the simple interpretation of the seemingly complex intensity patterns shown in Fig. 6 is that the probability of observing low index [001] and its symmetrically equivalent planes is high. Statistical distributions represented on the entire sphere result in multiple peaks, which are symmetrically equivalent to each other, leading to dubious interpretations. However, the analysis would be simplified if the distribution were to be plotted only in the boundary plane FZ, as the peaks in the FZ are unique. More importantly, when the data is represented in the FZ, the projection of the sphere is along the disorientation axis. If the data is projected along any other axes (e.g. the usual cubic 〈100〉 axes), the symmetries will be skewed making the data difficult to interpret. Hence, in the opinion of the authors, that data may be presented intuitively if expressed only in the boundary plane FZ.

The five-parameter FZ as described in this work is completely valid for simple metallic systems with any Bravais lattice. However, if the crystal basis consists of multiple atoms, additional degrees of freedom, such as the translation of the boundary-plane along its normal vector, should be considered. In complex materials, which may consist of multiple atoms in the crystal basis or in cases where the inter-atomic interactions are covalent or ionic, GB structure-property relationships may not vary as smoothly as those indicated here for simple mono-atomic metallic systems. However, even in these complex crystal systems, the unique representation technique described here is a necessary starting point for correlating crystallography with GB properties.

As evidenced by this work and work by others, GB plane orientations play an important role in defining a material’s properties. Simple classification by CSL or misorientation alone is problematic because properties, such as GB energy and mobility, vary by a large degree depending upon the GB plane orientation. The standardized representation presented here provides an intuitive way to interpret results and captures the natural symmetries of the GB space. Structure-property relationships naturally emerge as a result of this representation and these relationships provide promising insights into GB energy, excess free volume and temperature-dependent mobility trends.

## Methods

All GBs and their properties examined in this work originate from the catalogue of 388 Nickel GBs created by Olmsted *et al*.^33^ The catalogue of GBs is defined by all possible GBs whose boundary interface is periodic within a maximum box size of L_max _= 15 a_o_/2, where a_o_ represents the FCC lattice parameter. Among the 388 boundaries, there are 72 unique misorientations, from which numerous boundaries have been constructed by varying the GB plane orientation. The Nickel GBs were simulated using the Foiles-Hoyt embedded atom method (EAM) potential^44^ and represent the minimum energy configuration based on the construction described by Olmsted *et al*.^33^ In short, this construction finds the minimum energy structure of the GB by examining possible combinations of relative shifts between the two crystals, placement of the GB plane, allowed proximity of atoms from the two crystals, and removal of atoms from one crystal or the other when the allowed proximity of atoms from the two crystals is exceeded.

The property values for the catalogue of 388 GBs have been reported previously and follow standard calculation methods for energy and excess volume per unit GB area^33^ and temperature-dependent mobility trends^45^, which utilizes the synthetic driving force method^46^. It is noted that although mobility was measured for various magnitudes of the synthetic driving force^38^, the temperature-dependent mobility trends utilize only the 0.010 eV/atom driving force data^45^. These temperature-dependent mobility trends cover a range of different responses measured over the temperature range 600–1400 K. The basic trends of mobility, *M*, are generally characterized into thermally activated 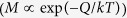, non-thermally activated (which can be further broken down into three trends: (i) athermal, *M = constant* or *dM/dT = *0, (ii) antithermal, 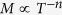, and (iii) thermally damped, 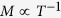), mixed modes of mobility (where different trends are exhibited over different temperature regimes, for example, thermally activated at low temperature and non-thermally activated at high temperature), immobile and finally GBs that exhibit unclassifiable mobility trends over the studied temperature range. For the purposes of this work, we characterize the mobility as thermally activated, non-thermally activated (including anti-thermal and thermally damped), athermal, immobile and “other” for the unclassifiable mobilities. For GBs that exhibited mixed-modes of mobility, only their most dominant mode is reported as one of the categories listed here.

## Additional Information

**How to cite this article**: Homer, E. R. *et al*. Grain Boundary Plane Orientation Fundamental Zones and Structure-Property Relationships. *Sci. Rep*. **5**, 15476; doi: 10.1038/srep15476 (2015).

## Supplementary Material

Supplementary Information

## References

[b1] Hall E. O. The Deformation and Ageing of Mild Steel: III Discussion of Results. Proc. Phys. Soc. B 64, 747–753 (1951).

[b2] Petch N. J. The Cleavage Strength of Polycrystals. J. Iron Steel Inst. 174, 25–28 (1953).

[b3] Hansen N. Hall–Petch relation and boundary strengthening. Scripta Mater. 51, 801–806 (2004).

[b4] Chiba A., Hanada S., Watanabe S., Abe T. & Obana T. Relation Between Ductility and Grain-Boundary Character Distributions in Ni_3_al. Acta Metall. Mater. 42, 1733–1738 (1994).

[b5] Fang T. H., Li W. L., Tao N. R. & Lu K. Revealing Extraordinary Intrinsic Tensile Plasticity in Gradient Nano-Grained Copper. Science 331, 1587–1590 (2011). 21330487 10.1126/science.1200177

[b6] Shimada M., Kokawa H., Wang Z. J., Sato Y. S. & Karibe I. Optimization of grain boundary character distribution for intergranular corrosion resistant 304 stainless steel by twin-induced grain boundary engineering. Acta Mater. 50, 2331–2341 (2002).

[b7] Lu L. Ultrahigh Strength and High Electrical Conductivity in Copper. Science 304, 422–426 (2004). 15031435 10.1126/science.1092905

[b8] Bagri A., Kim S.-P., Ruoff R. S. & Shenoy V. B. Thermal transport across Twin Grain Boundaries in Polycrystalline Graphene from Nonequilibrium Molecular Dynamics Simulations. Nano Lett. 11, 3917–3921 (2011). 21863804 10.1021/nl202118d

[b9] Meyers M. A., Mishra A. & Benson D. J. Mechanical properties of nanocrystalline materials. Prog. Mat. Sci. 51, 427–556 (2006).

[b10] Watanabe T. An approach to grain boundary design for strong and ductile polycrystals. Res. Mech. 11, 47–84 (1984).

[b11] Watanabe T., Tsurekawa S., Zhao X. & Zuo L. In Proceedings of the International Conference on Microstructure and Texture in Steels and Other Materials (eds. Haldar A., Suwas S. & Bhattacharjee D.) 43–82 (Springer London, 2009).

[b12] Randle V. Grain boundary engineering: an overview after 25 years. Mater. Sci. Tech.-Lond. 26, 253–261 (2010).

[b13] Randle V. & Jones R. Grain boundary plane distributions and single-step versus multiple-step grain boundary engineering. Mater. Sci. Eng. A 524, 134–142 (2009).

[b14] Schlegel S. M., Hopkins S. & Frary M. Effect of grain boundary engineering on microstructural stability during annealing. Scripta Mater. 61, 88–91 (2009).

[b15] Randle V. & Coleman M. A study of low-strain and medium-strain grain boundary engineering. Acta Mater. 57, 3410–3421 (2009).

[b16] Engelberg D. L., Newman R. C. & Marrow T. J. Effect of thermomechanical process history on grain boundary control in an austenitic stainless steel. Scripta Mater. 59, 554–557 (2008).

[b17] Palumbo G., Lehockey E. & Lin P. Applications for grain boundary engineered materials. JOM 50, 40–43 (1998).

[b18] Palumbo G., King P. J., Aust K. T., Erb U. & Lichtenberger P. C. Grain boundary design and control for intergranular stress-corrosion resistance. Scripta Metall. Mater. 25, 1775–1780 (1991).

[b19] Lehockey E. M. . On improving the corrosion and growth resistance of positive Pb-acid battery grids by grain boundary engineering. J. Power Sources 78, 79–83 (1999).

[b20] Tan L., Sridharan K., Allen T. R., Nanstad R. K. & McClintock D. A. Microstructure tailoring for property improvements by grain boundary engineering. J. Nucl. Mater. 374, 270–280 (2008).

[b21] Tan L., Sridharan K. & Allen T. R. Effect of thermomechanical processing on grain boundary character distribution of a Ni-based superalloy. J. Nucl. Mater. 371, 171–175 (2007).

[b22] Patala S., Mason J. K. & Schuh C. A. Improved representations of misorientation information for grain boundary science and engineering. Prog. Mater. Sci. 57, 1383–1425 (2012).

[b23] Materials Interfaces: Atomic-level structure and properties (eds Wolf D. & Yip S.), (Chapman & Hall, 1992).

[b24] Dehoff R. R. . Site specific control of crystallographic grain orientation through electron beam additive manufacturing. Mater. Sci. Tech.-Lond. 31, 931–938 (2015).

[b25] Johnson O. K. & Schuh C. A. The uncorrelated triple junction distribution function: Towards grain boundary network design. Acta Mater. 61, 2863–2873 (2013).

[b26] Barr C. M. . Anisotropic radiation-induced segregation in 316L austenitic stainless steel with grain boundary character. Acta Mater. 67, 145–155 (2014).

[b27] Matsunaga K. . HRTEM study on grain boundary atomic structures related to the sliding behavior in alumina bicrystals. Appl. Surf. Sci. 241, 75–79 (2005).

[b28] Han W. Z., Demkowicz M. J., Fu E. G., Wang Y. Q. & Misra A. Effect of grain boundary character on sink efficiency. Acta Mater. 60, 6341–6351 (2012).

[b29] D’Antuono D. S., Gaies J., Golumbfskie W. & Taheri M. L. Grain boundary misorientation dependence of beta phase precipitation in an Al-Mg alloy. Scripta Mater. 76, 81–84 (2014).

[b30] King A., Johnson G., Engelberg D., Ludwig W. & Marrow J. Observations of intergranular stress corrosion cracking in a grain-mapped polycrystal. Science 321, 382–385 (2008). 18635797 10.1126/science.1156211

[b31] Patala S. & Schuh C. A. Symmetries in the representation of grain boundary-plane distributions. Philos Mag 93, 524–573 (2013).

[b32] Pond R. C. & Vlachavas D. S. Bicrystallography. P. Roy. Soc. Lond. A Mat. 386, 95–143 (1983).

[b33] Olmsted D. L., Foiles S. M. & Holm E. A. Survey of computed grain boundary properties in face-centered cubic metals: I. Grain boundary energy. Acta Mater. 57, 3694–3703 (2009).

[b34] Frost H. J., Ashby M. F. & Spaepen F. A Catalogue of [100], [110], and [111] Symmetric Tilt Boundaries in Face-Centered Cubic Hard Sphere Crystals. Harvard Division of Applied Sciences 1–216 (1982).

[b35] Homer E. R., Foiles S. M., Holm E. A. & Olmsted D. L. Phenomenology of shear-coupled grain boundary motion in symmetric tilt and general grain boundaries. Acta Mater. 61, 1048–1060 (2013).

[b36] Bulatov V. V., Reed B. W. & Kumar M. Grain boundary energy function for fcc metals. Acta Mater. 65, 161–175 (2014).

[b37] Sutton A. P. & Balluffi R. W. Overview no. 61 On geometric criteria for low interfacial energy. Acta Metall. Mater. 35, 2177–2201 (1987).

[b38] Olmsted D. L., Holm E. A. & Foiles S. M. Survey of computed grain boundary properties in face-centered cubic metals-II: Grain boundary mobility. Acta Mater. 57, 3704–3713 (2009).

[b39] Gottstein G. & Shvindlerman L. S. Grain Boundary Migration in Metals. (CRC Press, 2010).

[b40] Cahn J. W., Mishin Y. & Suzuki A. Coupling grain boundary motion to shear deformation. Acta Mater. 54, 4953–4975 (2006).

[b41] Cahn J. W., Mishin Y. & Suzuki A. Duality of dislocation content of grain boundaries. Philos. Mag. 86, 3965–3980 (2006).

[b42] Rohrer G. S. . The distribution of internal interfaces in polycrystals. Z. Metallkde. 95, 197–214 (2004).

[b43] Patala S. & Schuh C. A. Representation of single-axis grain boundary functions. Acta Mater. 61, 3068–3081 (2013).

[b44] Foiles S. M. & Hoyt J. J. Computation of grain boundary stiffness and mobility from boundary fluctuations. Acta Mater. 54, 3351–3357 (2006).

[b45] Homer E. R., Holm E. A., Foiles S. M. & Olmsted D. L. Trends in grain boundary mobility: Survey of motion mechanisms. JOM 66, 114–120 (2014).

[b46] Janssens K. G. . Computing the mobility of grain boundaries. Nat. Mater. 5, 124–127 (2006). 16400330 10.1038/nmat1559

