# Supplementary material for: Grain Boundary Plane Orientation Fundamental Zones and Structure-Property Relationships
Source: Sci Rep. 2015 Oct 26;5:15476. doi: 10.1038/srep15476 (PMC4620447; doi:10.1038/srep15476)
Supplement: Supplementary Information [file srep15476-s1.pdf]

Supplementary Information: Grain Boundary Plane Orientation Fundamental Zones and Structure-Property Relationships

Eric Homer[1*], Srikanth Patala[2], Jonathan Priedeman[1]
[1] Department of Mechanical Engineering, Brigham Young University, Provo, UT 84602, USA.
[2] Department of Materials Science & Engineering, North Carolina State University, Raleigh, NC 27695, USA.

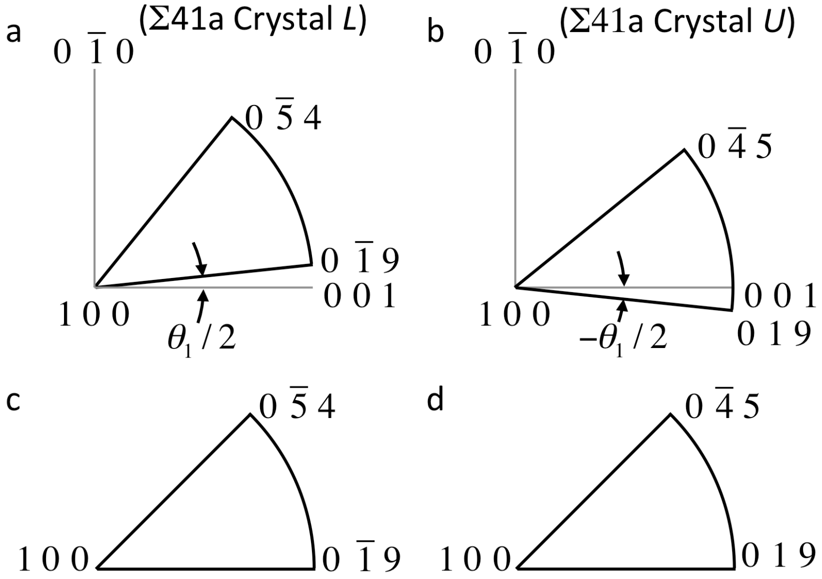

Figure S1 | Redundant Fundamental Zones of a Bicrystal. This figure illustrates how boundary plane orientation FZs in the two crystals are related, but are redundant since only a single orientation is required to describe any given GB. The example contained in this figure is for a Σ41a GB, where the FZ for crystals *L* and *U* are shown in (a) and (b), respectively. The FZs are separated by the rotation equal to the disorientation angle θ_1. The pure twist boundary about the disorientation axis [100] will have the same normal in both crystal *L* and *U*. The symmetric tilt normals will be nearly identical (related by a simple permutation of the indices); they are not identical as in the traditional case of a symmetric tilt as a result of the original definition of the axes. The FZs are rotated in (c) and (d) to standardize representation and to emphasize the fact that these two FZs do overlap when considering a spherical GB. This standardized representation is convenient because FZs of identical disorientation axes appear similar. However, the permutation of indices for the vertices of the FZ may suggest more similarity between the two regions than really exists. The indices of boundary planes inside the two FZs will not be related by simple permutation of the indices. However, the fact that the two FZs are related by a simple rotation equal to the disorientation angle means that all normals in crystal *U* are simply related to the normals in crystal *L* by a rotation equal to the disorientation angle. While the influence of the normal in crystal *U* may be important to properties, its actual value is redundant when providing a unique description of a GB. Therefore, for the standardized representations provided in this article, the boundary plane orientation as defined in the second crystal is not provided.

---

* Corresponding author: eric.homer@byu.edu
